# The relationship between cadence decline, cardiovascular drift and aerobic decoupling as a marker of fatigue in well trained cyclists

**DOI:** 10.1186/s13102-026-01678-w

**Published:** 2026-04-02

**Authors:** Artur Barsumyan, Christian Soost, Jan Adriaan Graw, Rene Burchard

**Affiliations:** 1 Philipps-Universität Marburg, Marburg, Germany; 2 Faculty III: Statistic and Econometrics, University of Siegen, Siegen, Germany; 3 Department of Anaesthesiology, Friedrich-Alexander-Universität Erlangen-Nürnberg (FAU), Universitätsklinikum Erlangen, Erlangen, Germany; 4 Department of Orthopaedics and Traumatology, University Hospital of Giessen and Marburg, Marburg, Germany; 5 Department of Orthopaedics and Trauma Surgery, Sports Medicine and Joint Centre, Lahn-Dill-Kliniken, Wetzlar, Dillenburg Germany; 6 Department of Orthopaedics and Trauma Surgery, c/o Sports Medicine and Joint Centre, Lahn-Dill-Kliniken, Rotebergstr. 2, Dillenburg, 35683 Germany

**Keywords:** Aerobic decoupling, Cardiovascular drift, Cadence, Cycling

## Abstract

**Background:**

Prolonged steady-state cycling is characterised by gradual neuromuscular and metabolic acute fatigue, which may affect an athlete’s movement patterns. We hypothesize that athletes might unconsciously reduce cadence as a compensatory strategy to maintain power output. To test this theory, we examined changes in cadence and internal load during extended submaximal cycling.

**Methods:**

To test this theory, 17 trained cyclists performed a monthly standardised 60-minute effort at 75% of their functional threshold power for five months, yielding 85 paired observations. Cadence behaviour was analysed alongside cardiovascular drift and aerobic decoupling in order to ascertain whether cadence decline reflects a surrogate marker of acute fatigue.

**Results:**

The results showed that cadence decline in the second half of the test was significantly correlated with both, cardiovascular drift and aerobic decoupling. Linear mixed model regression analysis revealed a robust association between cadence decline and cardiovascular drift (b = 0.61, *p* = 0.024), and a repeated measures correlation of *r* = 0.40 (*p* < 0.001). On average, each additional rpm of cadence decline corresponded to a 0.61% increase in cardiovascular drift. The correlation between cadence decline and aerobic decoupling was also significant (*r* = 0.38, *p* = 0.001) and the regression analysis shows that each additional rpm of cadence decline corresponds to a 0.58% increase in aerobic decoupling (b = 0.58, *p* = 0.007).

**Conclusion:**

These findings suggest that cadence decline is linked to both, cardiovascular and mechanical manifestations of acute fatigue. In practice, cadence monitoring offers a simple, non-invasive and widely accessible method of tracking fatigue. Moreover, it allows the design of training plans incorporating cadence-strategies and providing real-time feedback when cardiovascular strain may impair performance.

## Introduction

Cycling performance is a complex interplay of physiological, biomechanical, and neuromuscular factors [1]. Among these, cadence—the number of pedal revolutions per minute (rpm)—plays a critical role in how effectively power is generated and sustained during exercise [2]. Traditionally, cadence is considered a controllable variable that athletes can adjust to optimize efficiency and reduce muscular strain [3, 4]. However, even under controlled power output conditions, subtle variations in cadence may occur as a natural response to acute fatigue [5].

Cadence is a fundamental aspect of cycling performance and energy management. It reflects the balance between neuromuscular control and metabolic efficiency during sustained efforts [6, 7]. Although power output is usually the main external metric used to monitor endurance cycling, cadence can offer extra information about internal load and peripheral fatigue development, particularly under submaximal steady-state conditions [8].

Cadence is a primary driver of cycling power output, because power equals torque multiplied pedaling rate (rpm) [9]. When cadence decreases, torque requirements increase to maintain the same power level, potentially accelerating acute muscular fatigue [10]. Optimizing cadence, therefore, is critical for balancing muscular load and cardiovascular effort, ultimately impacting cycling performance.

Importantly, in both, training and testing settings, athletes typically perform at a cadence they have chosen themselves — they select a pedalling rate that they personally perceive as the most comfortable and sustainable for the given workload [11]. For experienced cyclists, this self-selected cadence is often close to the economical and optimal cadence, minimizing acute peripheral muscular fatigue at a given power output regardless of cycling conditions [12, 13]. In this context, changes in cadence over time may serve as a non-invasive marker of peripheral fatigue, indicating an imbalance between muscular and metabolic demands [14].

Although cadence, torque and acute fatigue are theoretically interconnected, there is little empirical evidence to support these relationships [15]. Most existing studies have examined torque using instrumented cranks or ergometers, which are rarely feasible in everyday training environments. Furthermore, these investigations have predominantly focused on acute fatigue responses during isolated sessions, often in high-intensity or sprint-based cycling disciplines [6, 16, 17]. Consequently, our understanding of how cadence adjusts during prolonged submaximal efforts and how these adaptations reflect fatigue development over time is limited. However, endurance cycling is characterised by sustained workloads that trigger various physiological responses, such as shifts in plasma volume, dehydration and environmental stress. These responses may interact with fatigue-related changes in cadence. One such response is cardiovascular drift, which is defined as a gradual increase in heart rate despite a constant external workload. This phenomenon commonly occurs alongside aerobic decoupling, reflecting a dissociation between internal (heart rate) and external (power output) load [18–20].

As fatigue accumulates during prolonged steady-state exercise, we hypothesize that cyclists unconsciously adjust their pedalling cadence in an attempt to compensate for acute fatigue. We expect that athletes decrease the pedalling cadence with increased acute fatigue and consecutively increase torque per pedal stroke to maintain power output. Consistent observations of cadence decline during multiple repeatable and controllable efforts may provide meaningful insight into an athlete’s peripheral fatigue profile. This study aims to document and analyse the cadence behaviour of trained cyclists during a standardized 60-minute effort at 75% of functional threshold power (FTP), conducted monthly over a five-month period. By examining intra-individual patterns and their relationship with cardiovascular drift and aerobic decoupling, the study will explore whether cadence decline may serve as a marker of acute fatigue-related adaptation and altered performance regulation.

## Methods

### Participants and data collection

Seventeen male cyclists (*N* = 17; age 31 ± 3.3 years; body weight 76,2 ± 4,2 kg; initial absolute FTP 243,8 ± 27,9 W; 4,3 ± 1,09 years in endurance sport; weekly training volume 6,38 ± 2,3 h·week^-1^) were enrolled in this study. Each participant had a minimum of four years of consistent endurance training, mainly focusing on road and gravel cycling. The cyclists were recruited through local cycling clubs and personal networks, forming a convenience sample of experienced, competitive athletes. All participants gave written informed consent to participate in this study. This study was conducted in accordance with the Declaration of Helsinki (2013) and the ethics committee of the Philipps University of Marburg approved the study (24–327 RS). The study was not formally registered. The data are available on reasoned request to the corresponding author. Due to participant confidentiality, institutional data protection policies and the ethical approval framework under which the data were collected, restrictions apply.

### Testing protocol

Each participant performed a standardised cycling protocol once per month over a five-month period. These assessments were incorporated as structured sessions within the athletes’ regular training schedules. The protocol comprised a 10-minute incremental warm-up, increasing from 55% to 65% of the individual’s current FTP, followed by a 60-minute steady-state bout at 75% FTP.FTP values were validated and adjusted monthly using WKO 5 cycling analytics software (version 5.90, Peaksware LLC, Lafayette, Colorado, USA), thereby ensuring that the test intensity remained aligned with each cyclist’s current fitness level. As all subjects were experienced cyclists accustomed to cadence variation in training, a dedicated familiarisation session was deemed unnecessary.

Cardiovascular drift may be influenced by numerous factors, including hydration status, circadian rhythms, environmental conditions and nutritional state [21]. Consequently, a rigorous, standardised testing protocol was applied to all participants. Test sessions were conducted at a consistent time of day in a controlled indoor setting with adequate ventilation and directed fan cooling towards the torso. Fluid intake was standardised at 500 ml per hour, consisting of water or an electrolyte solution. Subjects abstained from food during testing and were instructed to consume a standardised meal two hours beforehand, eliminate caffeine within four hours of the test and avoid alcohol and strenuous physical activity for 24 h prior to testing. Outside of testing, participants adhered to their regular, coach-prescribed training regimens. Training content and load were not standardised to ensure that physiological responses reflected authentic training conditions.

Experimental sessions were performed using the participants’ personal racing bicycles, which were mounted on electromagnetically braked, direct-drive cycling trainers (Kickr v5, Wahoo Fitness, Atlanta, USA or Tacx, Wassenaar, Netherlands). These were calibrated according to the manufacturers’ recommendations to ensure data reliability. Power output was continuously measured using Garmin Rally (Garmin Ltd., Olathe, Kansas, USA) or Favero Assioma (Favero Electronics, Treviso, Italy) power meters paired with a head unit. A zero-offset calibration was completed before each session in accordance with the manufacturers’ guidance. Heart rate was recorded throughout all tests via a chest strap monitor (Garmin HRM-Pro, Garmin Ltd., Olathe, Kansas, USA) linked to the portable cycling computer.

### Data analysis

Two independent researchers performed and analysed the data collection, including inspection for errors, using the commercially available cycling software TrainingPeaks (version 9.3.0, Peaksware LLC, Lafayette, CO, USA) and WKO 5. No data imputation was performed, and only complete and error-free experimental sessions were included in the evaluation. The variables extracted for each test included FTP [watt], heart rate, HR [bpm], cadence [rpm], average power output [watt], cardiovascular drift [%], aerobic decoupling [%].

Cardiovascular drift and aerobic decoupling were quantified using standardized, replicable definitions. For both metrics, the 60-min test was divided into two equal epochs: the first half (0–30 min) and the second half (31–60 min). Cardiovascular drift was calculated using heart rate only, expressed as the percentage change from the first half to the second half of the ride and was calculated using the following Eq. 1:

1 $$\:Cardiac\:Drift\:\left(\%\right)=\frac{HR\left(Second\:Half\right)\:-\:HR\left(First\:Half\right)}{HR\:\left(First\:Half\right)}x100$$

Aerobic decoupling (Pw: HR decoupling) was calculated using the ratio of power to heart rate, comparing the first and second halves of the test. A greater percentage of Aerobic decoupling indicates a stronger divergence between external (power) and internal (heart rate) load. Decoupling was expressed as the relative change in Pw: HR between epochs and calculated using the following Eq. 2:

2 $$\:Pw:Hr\:Decoupling\:\left(\%\right)\frac{Pw:Hr\:\left(First\:Half\right)-\:Pw:Hr\:\left(Second\:Half\right)}{Pw:Hr\:\left(First\:Half\right)}x100$$

### Statistical analysis

Data analysis was performed using R (R Core Team, Vienna, Austria). Associations between cardiovascular drift and cadence decline as well as between aerobic decoupling and cadence decline were evaluated across different test modalities using repeated measures correlation with the rmcorr package. Repeated-measures correlation (rmcorr) estimates the linear association between two variables within individuals by removing all between-subject differences in level and focusing solely on the covariation of deviations from each person’s mean. In practice, rmcorr fits parallel regression lines with a common slope, thereby accounting for the non-independence of repeated observations and isolating the within-subject relationship from between-subject variability [22]. This yields an adjusted correlation coefficient that reflects a consistent association across individuals. In addition, we estimated simple linear mixed models (random intercept model) using cluster robust standard errors (CR2 from the clubSandwich package) to corroborate these associations, as mixed models flexibly account for inter-individual variability in intercepts and slopes and provide a robustness check beyond the correlation-based approach. The level of statistical significance was set to *p* < 0.05.

## Results

Cadence declined significantly from the first to the second half of the test (Δrpm = − 1.75 rpm, 95% CI − 2.01 to − 1.50, *p* < 0.001), Table 1.

**Table 1:** Comparison of cadence between the first (0–30 min) and second half (31–60 min) of the cycling test

Measured parameter	Mean ± SD	Mean Δrpm (95% CI of Δrpm)	Effect size (Cohen’s d)	Paired t-test
Cadence in first half (0–30 min)	86,6 ± 3,47	–1.75 rpm (–2.01 to − 1.50 rpm)	1,49	T = 13,76; *p* < 0,001
Cadence in second half (31–60 min)	84,8 ± 3,47			

Mean cardiovascular drift was found to be 2.09 ± 2.29%, while the mean change in aerobic decoupling was found to be comparable at 2.00 ± 2.38%. Regression analysis indicated that cadence drop was positively associated with both cardiovascular drift and aerobic decoupling, Table 2.

**Table 2:** Regression results linking cadence drop with cardiovascular drift and decoupling

	Dependent variable:	
	Cardiovascular Drift %	Aerobic Decoupling %
	(1)	(2)
Cadence decline_rpm	0.61^*^ (0.24)	0.58^**^ (0.18)
	[0.10;1.13]	[0.19;0.97]
Constant	1.02 (0.67)	0.99 (0.66)
	[-0.42;2.45]	[-0.43;2.41]
Observations	85	85
Log Likelihood	-167.097	-170.252
Akaike Inf. Crit.	342.194	348.504
Bayesian Inf. Crit.	351.964	358.275

Note ^*^p < 0.05; ^**^p < 0.02; ^***^p < 0.001

Table 3 summarizes the repeated measures correlations among the three key fatigue indicators: cadence decline (the difference in mean pedalling rate between minutes 0–30 vs. 31–60), cardiovascular drift, and aerobic decoupling.

**Table 3:** Repeated measures correlations

Measured pair	*r* (95% CI)	*p*-value
Cadence decline vs. cardiovascular drift (%)	0.40 (0.15–0.60)	*p* < 0,001
Cadence decline vs. aerobic decoupling (%)	0.38 (0.15–0.57)	*p* < 0,01

A higher decline in cadence during the second half of the test is significantly correlated with both higher cardiovascular drift and higher aerobic decoupling. The correlation is moderate and significant for both relationships.

**Fig. 1:**
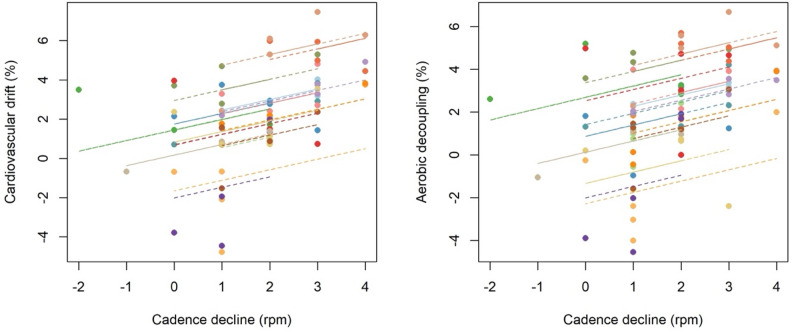
Relationship between cadence decline (rpm) and cardiovascular drift (left) as well as aerobic decoupling (right). Individual regression lines are displayed for each participant, illustrating within-subject associations

The linear mixed model revealed a significant positive association between cadence decline, cardiovascular drift and aerobic decoupling (Fig. 1).

Specifically, for each additional rpm decrease in cadence (rpm), cardiovascular drift increased on average by 0.61% points (b = 0.61, SE = 0.24, [0.10;1.13], *p* = 0.024), after accounting for the clustering of repeated measurements within athletes. This indicates that greater reductions in cadence were systematically related to stronger cardiovascular drift, independent of between-athlete differences. In addition, a significant positive effect was observed for aerobic decoupling: each additional rpm decrease in cadence was associated with an average increase of 0.58% points in decoupling (b = 0.58, SE = 0.18, [0.19;0.97], *p* = 0.007), suggesting that cadence decline is consistently linked to both cardiovascular drift and aerobic decoupling across athletes.

**Fig. 2:**
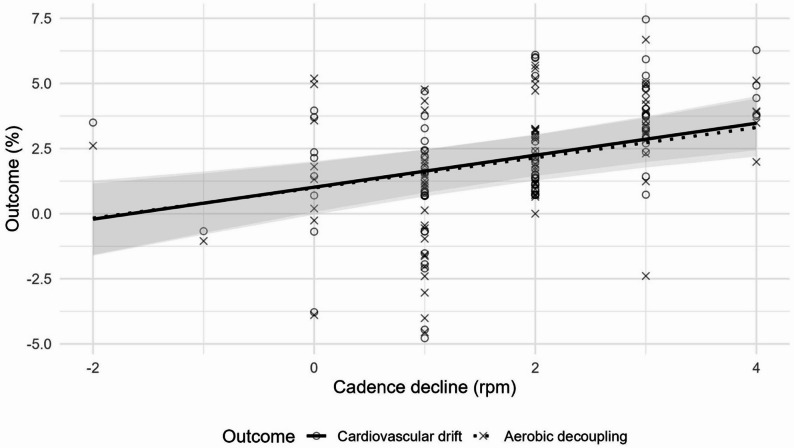
Predicted effects of the relationship between cadence drop (rpm) and cardiovascular drift (solid line) as well as aerobic decoupling (dotted line), with 95% confidence intervals (shaded area)

The repeated-measures correlations revealed moderate but significant associations between cadence decline and both cardiovascular drift (*r* = 0.40, *p* < 0.001) as well as aerobic decoupling (*r* = 0.38, *p* < 0.01) (Fig. 2).

These findings are in line with the results of the linear mixed models, which likewise indicated significant positive relationships: each additional 1 rpm decrease in cadence was associated with an average increase of 0.61% points in cardiovascular drift and 0.58% points in aerobic decoupling. Taken together, both the correlation and regression analyses consistently demonstrate that greater reductions in cadence are systematically linked to stronger cardiovascular drift and higher aerobic decoupling, underscoring the robustness of this relationship across athletes.

## Discussion

This study shows, that even during relatively short efforts, such as a one-hour cycling test at 75% of an athlete’s FTP, it has been observed that cadence tends to decline over time. Our data, collected from amateur cyclists during monthly test sessions over a period of five months, consistently show that most of athletes pedal at a higher cadence during the first 30 min of the test compared to the final 30 min. This decline in cadence, typically ranging from one to five rpm, suggests that athletes may inadvertently adjust their pedalling rate when acute fatigue develops. This decline in cadence, which typically ranges from one to five rpm, suggests that athletes may adjust their pedalling rate unintentionally as they become fatigued. One possible explanation is that the reduction in cadence reflects an unconscious adjustment to pacing or a strategy to reduce perceived exertion rather than being purely a mechanical necessity. Consequently, cyclists may compensate by applying greater force to each pedal stroke in order to maintain the same power output. However, the precise physiological or psychological drivers of cadence reduction remain unclear. Hypothetically, this mechanical shift may be an unconscious compensatory mechanism, whereby cyclists maintain the required power output by increasing pedal torque per stroke when muscular fatigue or neuromuscular inefficiency limit their ability to sustain higher cadences. However, to prove this physiological mechanism, measurements of torque and neuromuscular function would be needed. As our study did not include direct physiological or biomechanical measurements, such as torque output, electromyography or cardiometabolic data, the mechanisms underlying cadence decline cannot be inferred from our results. Therefore, in this context, cadence behaviour should be interpreted as a mechanical response correlated with cardiovascular drift and aerobic decoupling, rather than as direct evidence of peripheral fatigue.

Our findings demonstrate that cyclists’ loss of pedalling cadence in the second half of a 60-minute steady test is associated with two established fatigue markers: cardiovascular drift and power–heart rate decoupling. Cardiovascular drift shows a stronger and more explanatory association. The correlation (*r* ≈ 0.40) between decline in cadence and cardiovascular drift indicates a robust link: athletes who show greater cardiovascular drift also tend to exhibit steeper declines in cadence when the test progresses. Although aerobic decoupling shows a positive correlation (*r* ≈ 0.38), this does not provide any further insight that is not already explained by cardiovascular drift.

From a physiological perspective, cardiovascular drift reflects progressive cardiovascular strain, which manifests as an increased heart rate and decline in stroke volume at a stable power output [23]. In addition, it has been hypothesized that cardiovascular drift may serve as a mechanism, mitigating potential damage from prolonged, high-intensity myocardial contraction [18]. Our results support this understanding by demonstrating that as a concurrent mechanical adjustment the pedalling cadence is reduced. This mechanical shift may be an unconscious compensatory mechanism, whereby cyclists maintain the required power output by increasing pedal torque per stroke when muscular fatigue or neuromuscular inefficiency limit their ability to sustain higher cadences [9, 24].

Cadence modulation is a behavioural response to acute fatigue mechanisms. A higher cadence (e.g. 90–100 rpm) reduces the torque required for each pedal stroke, spreading the mechanical stress over a greater number of contractions and thus delaying peripheral fatigue [1, 25]. However, this strategy increases cardiovascular strain due to an elevated heart rate and oxygen uptake [26]. Conversely, a lower cadence (e.g. 70–80 rpm) reduces the metabolic cost, but increases the torque requirements and accelerates acute muscle fatigue [15]. Therefore, the decline in cadence observed in the second half of an exercise session may represent a fatigue-induced trade-off: as metabolic reserves dwindle and peripheral fatigue accumulates, athletes unconsciously reduce their cadence to mitigate cardiovascular strain. This results in greater torque demands and localized muscle fatigue [27]. Recent research has shown that these changes signal accumulating physiological stress that may compromise performance and efficiency over time [28]. However, while these adaptations are recognized, considerably less attention has been given to how they might influence or coincide with biomechanical factors, such as cadence modulation. Exploring these intersections is crucial for a comprehensive understanding of fatigue mechanisms and for optimizing pacing strategies during prolonged exercise [29].

Acute fatigue can reduce the efficiency of muscle contractions. A higher force per pedal stroke is required despite a stable power output [30]. When fatigue progresses, the focus shifts from metabolic efficiency to strength endurance [16]. The inverse relationship between torque and cadence at a constant power output creates a biomechanical feedback loop with critical implications for fatigue management. The inverse relationship between torque and cadence at a constant power output establishes a biomechanical feedback loop that has significant implications for managing fatigue. Consequently, a reduction in cadence can create a vicious cycle whereby fatigue causes a decline in cadence, thereby increasing torque and accelerating fatigue [31]. This cycle is particularly consequential in events requiring sustained power outputs, such as time trials and long climbs, where premature cadence shifts could hasten exhaustion.

Despite the moderate associations observed, a decline in cadence alone cannot fully account for the variance in cardiovascular drift or aerobic decoupling. The residual heterogeneity emphasizes the multifactorial nature of fatigue in endurance exercise [32, 33]. Several factors, such as muscle fibre type, pacing strategy, hydration status, and environmental conditions, all may independently or interactively influence both, cardiovascular and mechanical responses [34]. Together, these factors suggest that decline in cadence should not be considered as the sole or universal marker of acute fatigue, but rather as one component within a broader, multidimensional framework. To gain an understanding of the complex interplay between cardiovascular and peripheral fatigue mechanisms, the decline in cadence should be considered alongside other physiological parameters such as heart rate and power output. A decline in cadence is an immediately observable marker of developing fatigue and may offer coaches and athletes a practical tool for real-time assessment of fatigue without the need to rely solely on heart-rate analysis.

### Limitations

A key limitation of this study is the absence of direct physiological or biomechanical markers of peripheral fatigue. Although a decline in cadence is considered as an indicator of fatigue progression, no concurrent torque measurements or surface electromyography were collected. Without these measures, we cannot definitively attribute cadence reductions to peripheral fatigue and must consider alternative explanations such as individual effort regulation, psychological factors, and biomechanical adjustments. Furthermore, comprehensive cardiometabolic data such as ECG signals, oxygen uptake, and minute ventilation were not recorded. Therefore, our ability to interpret contributions of the cardiometabolic system to the observed changes is restricted and limits the physiological resolution of our findings. Therefore, decline in cadence should be regarded as an indirect, observational marker rather than a mechanistic proof of neuromuscular impairment. This study did not include an a priori power analysis, and some analyses may therefore be underpowered to detect small effects. Instead of relying on post-hoc power calculations, we report effect sizes together with their confidence intervals, which more appropriately describe the precision and uncertainty of the estimates.

## Conclusion

From a practical perspective, monitoring cadence may offer a simple, non-invasive way to track mechanical changes associated with increasing cardiovascular strain during prolonged cycling. A progressive reduction in cadence may indicate a behavioural response that correlates with heart rate drift and aerobic decoupling rather than being direct evidence of peripheral fatigue. As cadence can be easily measured using cycling devices, it is a useful metric for athletes and coaches to track changes in fatigue in real time. Beyond immediate monitoring, cadence data could also support training design. Our findings suggest that integrating cadence data with established fatigue indicators, such as heart rate and power output decline, might have a potential to improve the assessment of fatigue during submaximal cycling. However, future studies incorporating direct neuromuscular and cardiometabolic measurements are needed to clarify the physiological mechanisms underlying cadence decline, and to determine practical thresholds for training and performance monitoring.

## Data Availability

Data are available on reasonable request to the corresponding author.

## Abbreviations

FTP: Functional threshold power
Rpm: Revolutions per minute
CI: Confidence interval
SE: Standard error
